# The views of general practitioners and practice nurses towards the barriers and facilitators of proactive, internet-based chlamydia screening for reaching young heterosexual men

**DOI:** 10.1186/1471-2296-15-127

**Published:** 2014-06-27

**Authors:** Karen Lorimer, Susan Martin, Lisa M McDaid

**Affiliations:** 1 Institute for Applied Health Research, Glasgow Caledonian University, School of Health and Life Sciences, Cowcaddens Road, Glasgow G4 0BA, Scotland; 2 MRC/CSO Social and Public Health Sciences Unit, University of Glasgow, 200 Renfield Street, Glasgow G2 3QB, Scotland

## Abstract

**Background:**

Chlamydia trachomatis is a common bacterial sexually transmitted infection (STI), which disproportionately affects young people under 25 years. Commonly, more women are offered screening than men. This study obtained the views of general practitioners and practice nurses towards Internet-based screening and assessed levels of support for the development of proactive screening targeting young heterosexual men via the Internet.

**Methods:**

Semi-structured telephone interviews with 10 general practitioners and 8 practice nurses, across Central Scotland. Topics covered: experience of screening heterosexual men for chlamydia, views on the use of the Internet as a way to reach young men for chlamydia screening, beliefs about the potential barriers and facilitators to Internet-based screening. Transcripts from audio recordings were analysed with Framework Analysis, using QSR NVivo10.

**Results:**

Experiences of chlamydia screening were almost exclusively with women, driven by the nature of consultations and ease of raising sexual health issues with female patients; few practice nurses reported seeing men during consultations. All participants spoke in favour of Internet-based screening for young men. Participants reported ease of access and convenience as potential facilitators of an Internet-based approach but anonymity and confidentiality could be potential barriers and facilitators to the success of an Internet approach to screening. Concerns over practical issues as well as those pertaining to gender and socio-cultural issues were raised.

**Conclusions:**

Awareness of key barriers and facilitators, such as confidentiality, practicality and socio-cultural influences, will inform the development of an Internet-based approach to screening. However, this approach may have its limits in terms of being able to tackle wider social and cultural barriers, along with shifts in young people’s and health professionals’ attitudes towards screening. Nevertheless, employing innovative efforts as part of a multi-faceted approach is required to ensure effective interventions reach the policy agenda.

## Background

Chlamydia, the most common bacterial sexually transmitted infection (STI) in the UK [1], disproportionately affects young people under 25 years. Prevalence in the general population is mostly similar for women and men [2]. Screening for chlamydia among the target population at risk of infection can lead to early detection, reduction in transmission and to a reduction in associated morbidities [3]. Thus, early identification and treatment of infections remains paramount. There are two screening approaches: proactive, or systematic, which use population registers to invite members for a test, and; opportunistic, which involves health professionals offering tests to patients attending health care or other defined settings for unrelated reasons [4]. Various countries have taken an opportunistic approach to control the population prevalence of chlamydia including England, which has a National Chlamydia Screening Programme (NCSP). A randomised controlled trial of opportunistic screening is underway in Australia, with results from the ACCEPt trial due in 2014 [5]. Norway is exploring and planning a proactive approach [6], and a recent trial conducted in three regions of the Netherlands (the Chlamydia Screening Implementation programme) [7], evaluated the effectiveness of systematic, yearly chlamydia screening. The Dutch trial found no impact on chlamydia positivity rates or on estimated population prevalence [7].

Whilst treating infections remains paramount, opportunistic approaches have largely failed to demonstrate sufficient coverage among the target population [8], have tended not to achieve sustained screening engagement over time or show effectiveness in reducing population prevalence [9]. It is also an approach which has thus far largely failed to include men to the same extent as women: the NCSP in England reached only 16% of young people aged 15–24 years (24% of women and only 8% of men) in 2007/08 [3], although some areas have since seen higher coverage. In Scotland, in 2010, 27% of all tests performed were on men [10]. Screening men is primary prevention for women and could help normalise screening and reduce the psychosocial stigma for women associated with submitting one sex to surveillance, testing and treatment [11]. However, barriers to a proactive approach include: the largely asymptomatic nature of the infection which provides no physical cue with which to seek healthcare; and the poor willingness among young people to access ‘stigmatising’ genitourinary medicine (GUM) or other clinical settings [12]. There is a continued need to evaluate different approaches to screening, paying attention to the involvement of young adult men.

Online social media, such as social networking sites (e.g., Facebook), blogs and chat rooms have become integral parts of adolescents' and young adults' lives. Interactive computer-based interventions for sexual health promotion were assessed in a systematic review and found to be effective tools for learning about sexual health, and showed positive effects on self-efficacy, intention and sexual behaviour [13]. Computer-based technology has also been effective in increasing condom use for HIV prevention [14]. Media such as the Internet offers exciting potential for sexual health interventions, as they can be a low cost and flexible way to reach young people and could provide the easy, convenient and confidential approach to screening that young people report they want [12]. Young people hold favourable views towards the use of technology for STI screening [15-17], want straightforward information [15], authenticity of voice on websites [18] and to be treated like adults [17]. Postal testing kits, obtained via the Internet are acceptable [19], but direct mailing of kits appear to perform better than test-request kits [20]. Internet-based approaches are also showing better screening uptake than clinic-based approaches among men [21]. The use of the Internet for chlamydia screening has the potential to ease pressure from time-limited staff, such as general practitioners (GPs) and practice nurses (PNs), and in contexts where there is the absence of a national programme or where there may be limited availability of screening outwith specialist sexual health services, the Internet could fill a gap to act as an adjunct to clinical services. Whilst there remain challenges in building a sufficiently robust evidence-base on which to devise screening policy [9], further research questions continue to be posed, including whether sustaining a certain level of uptake with repeat systematic screening could lead to a reduction in chlamydia prevalence [7].

The intent of this study was to gather evidence to inform the subsequent design of an Internet-based approach to chlamydia screening targeting young men (aged 16–24 years). Understanding the views of GPs and PNs to the potential use of registers to contact men for chlamydia screening is vital to the future design of a randomised controlled trial (RCT) involving patient lists. Further, it is vital to understand the acceptability amongst primary care professionals regarding potential increased workloads from a new approach which may drive patients towards primary health care [22], particularly in a context where there is no existing screening programme or consistent culture of screening for chlamydia. Thus, to aid the development of our intervention, we explored the barriers and facilitators to implementing an Internet chlamydia screening approach, including the acceptability of such an approach amongst young men and health professionals. Elsewhere we report men’s views from fifteen focus groups (n = 60) [17]; here we detail the views of the GPs and PNs.

## Methods

### Design and setting

Participants were selected purposefully, to include GPs and PNs working at practices across areas of low to high deprivation across two regions across central Scotland (known as the ‘central belt’ of Scotland, with Glasgow in the west through to the capital, Edinburgh, in the east). These regions contain cities with the two largest sexual health clinics as well as other hub services, falling under two major NHS Board areas; as such, we sought the views of staff working within these two key areas. We also sought practices with varying percentages of young men (aged 15–24 years) registered with the practice. This was to obtain views from professionals who *may* have different perspectives due to practice-based issues (e.g., serving a largely elderly population may not incline staff towards sexual and reproductive health services, including chlamydia screening). We set out to conduct short (around 30 minutes), focused semi-structured telephone interviews in order to generate explanations of the specific phenomena under consideration. We aimed to recruit twenty GPs and PNs (10 of each). To reach GPs and PNs, we sent 241 letters outlining the study to practices across the two chosen regions. In the letter we stated: ‘*We would appreciate your consideration of this invitation and will follow-up this letter with a telephone call*’. We then began to contact a purposive sample of these (seeking to try to include practices located within high and low deprivation areas as well as those with high and low percentages of young men registered) to try to include a plurality of voices and experiences; we continued this process until we had conducted eighteen interviews and had reached data saturation, whereby the same comments were being offered to questions with no new data emerging. At saturation we ceased contacting practices so most practices were not contacted with a follow-up telephone call. As such, we do not have a full response rate for the 241 letters sent.

To identify practices, we assigned general practices with a deprivation score by using data provided online by Information Statistics Division (ISD) Scotland (http://www.isdscotland.org/Health-Topics/General-Practice/Workforce-and-Practice-Populations/Practices-and-Their-Populations/) and referring to the Scottish Index of Multiple Deprivation (SIMD) quintiles, where 1 is the most deprived and 5 is the least deprived) to link the practice postcode data with SIMD quintile for that small area. The Scottish Government website provides an interactive map to identify the SIMD rank of small areas [23]. The 2012 SIMD combines 38 indicators across 7 domains, namely: income, employment, health, education, skills and training, housing, geographic access and crime; the overall index is a weighted sum of the seven domain scores. We were also able, using ISD data, to identify the percentage of men aged 15–24 years registered at the practice – although we note that the variation was limited, with 6% of practices having fewer than 10% of young men aged 15–24 registered at the practice and a handful having more than 45%, with the average being 13%. We therefore attempted to recruit the few practices with more extreme percentages. We offered remuneration for participants’ time (£30 for GPs and £20 for PNs).

### Data collection

**Figure 1:**
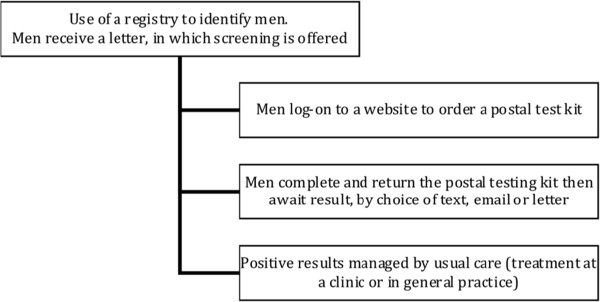
Process of internet-based proactive screening provided to the general practitioners and practice nurses.

Telephone interviews were designed to be brief, and lasted between 15 and 35 minutes, with most being around 30 minutes. The semi-structured topic guide was drawn from key areas to emerge from the focus groups with young men [17], including screening experiences, views towards the Internet-based approach and their identification of barriers and facilitators, as well as from literature on this topic [5,22,24] suggesting practitioner workload and training issues were affecting screening offers. The final topic guide focused on: experience of screening women and men (to give context to views); use of technologies within the practice around screening (for example, text results); views towards the use of the Internet as a way to reach young men for chlamydia screening; and views towards any barriers and facilitators to an Internet-based screening approach. Practice nurses were asked additional question about willingness to undertake partner notification to explore whether a future Internet approach could rely on general practice to deal with the follow-up of positives. The proposed approach as explained to GPs and PNs is illustrated in Figure 1.

### Data analysis

The telephone interviews were conducted by the first author (KL) and audio recorded, then transcribed intelligent verbatim and checked for accuracy. QSR Nvivo10 was used to facilitate analysis. Transcripts were read repeatedly by SM and a thematic coding framework was developed on a collaborative, iterative basis within the team (including SM, KL, LM), with discrepancies in early interpretation being fully discussed within the team and an agreement reached; the Framework Approach was employed, where data are coded, indexed and charted systematically, then organised using a matrix or framework [25]. There are five key stages of Framework: familiarisation, identifying a thematic framework, indexing, charting, mapping and interpretation. Framework Analysis begins deductively from the study aims and objectives (generating prepositions), but is also inductive (using patterns and associations derived from observations) [26]. SM indexed the data using the coding framework, with a third checked by KL, before data were ‘charted’ into the framework matrix. The charting and interpretation stage was conducted by SM with continued dialogue and checking of the data with KL. Constant comparison was carried out to check for deviant cases as well as similarities, in an iterative process; we explored whether there were any differences in experience and views by deprivation and percentage of young men registered to the practice attributes, as well as by the gender of the GP (but not for PN as we were only able to interview female PNs) and between the GPs and PNs. There were few differences in views between GPs and PNs based on the deprivation score and percentages of men registered at the practice, but we outline them where there are.

### Ethics

Ethical approval was obtained from West of Scotland Research Ethics Committee 1 (Ref: 11/AL/0398). Consent was obtained by participants being read the consent form over the telephone and verbally agreeing to each point. Participants were asked for this to be audio-recorded, so that a recorded record of the consent was obtained. A copy of the form was posted to the participants for reference. All participants agreed to the consent process and their interview being audio-recorded.

Illustrative quotes are used throughout indicating the participants’ category: GP for general practitioner; PN for Practice Nurse; and the gender, practice SIMD code and percentage of young men registered at the practice (e.g., PN1, Female, SIMD 1, 14.3%).

## Results

### Participant and practice demographics

We conducted telephone interviews with 10 GPs and 8 PNs between February and May 2012. Table 1 shows the spread of practices from SIMD quintiles (1 being most deprived and 5 being least deprived) and the percentages of young men aged 15–24 years registered with the practice. Whilst we recruited GPs and PNs from practices across SIMD categories, and we were able to recruit even numbers of male and female GPs to the study, we were unsuccessful in recruiting any male PNs, despite purposefully seeking them though searching the websites of practices and attempting ‘snowballing’ techniques. Across the eighteen practices the percentage of young men registered with the practice were broadly similar, with the exception of a few, which had either a very low percentage (e.g., 6.5%) or in one case a very high percentage (42.9%) (see Table 1); however, given many practices have similar percentages, this inclusion is not surprising.

**Table 1:** Participant and practice demographics

	**Gender**	**Practice SIMD quintile**	**Location (Glasgow/Edinburgh)**	**% 15–24 year old males registered**		**Gender**	**Practice SIMD quintile**	**Location (Glasgow/Edinburgh)**	**% 15–24 year old males registered**
GP1	Female	1	Glasgow	12.9	PN1	Female	1	Glasgow	14.3
GP2	Male	5	Edinburgh CHP	9.6	PN2	Female	5	Glasgow	6.5
GP3	Female	5	Edinburgh CHP	42.9	PN3	Female	3	Glasgow	12.2
GP4	Male	5	Edinburgh CHP	10.2	PN4	Female	2	Glasgow	10.1
GP5	Female	1	Glasgow	18.4	PN5	Female	2	Glasgow	10.5
GP6	Female	1	Glasgow	13.7	PN6	Female	5	Edinburgh CHP	12.8
GP7	Male	1	Glasgow	14.0	PN7	Female	5	Edinburgh CHP	13.9
GP8	Female	5	Edinburgh CHP	16.1	PN8	Female	2	Glasgow	12.0
GP9	Male	4	Edinburgh CHP	10.9					
GP10	Male	2	Edinburgh CHP	11.2					

### Experiences of screening women and men for chlamydia

General practitioners, and particularly the PNs, described their experiences of chlamydia screening as being almost exclusively with women, which reflects the national testing figures for Scotland showing that 73% of tests were conducted with women [10]. Participants perceived there to be a higher attendance at general practice by women compared to men, driven by contraception consultations, cervical smear tests (Pap tests) or breast screening, and this was cited regularly by both the GPs and PNs as underlying their perceived greater opportunity for, and thus experience of, opportunistic screening of women. Perceptions of low attendance by young men were cited by both GPs and PNs as a major reason for their lower experience of screening male patients for chlamydia. Practice Nurses, far more than the GPs, reported very few occasions of interacting with young men, even during times when screening was being encouraged by the Health Boards.

My experience of screening young men has probably been part of the opt-in enhanced service, that ran over, I think, a two-year period, and has now ceased. During that time, we were opportunistically asking people if they wanted to be screened, and that was people aged from 15 to 24, as I recall. During that time, I didn’t approach any young men, because I don’t think it’s an age group that I actually see very often, in my particular field. (PN3, Female, SIMD3, 12.2%)

The few occasions mentioned tended to be for specific clinic attendance, such as an asthma clinic, but such clinics were not consistently available across the practices represented by the participants as they depended on patient need across practices. GPs, who saw more men than the PNs, gave their views on why they did not screen as many men as women:

I suppose [pause] - I guess one of the reasons for the differences that we see more young female patients then we see young men, we have more interaction with them, they come in for their contraceptive pill and they generally consult more frequently. We don’t see that in many 20 year old men in and about the place so that would probably explain the difference in my testing rates between the two groups. (GP4, Male, SIMD5, 10.2%)

However, when participants were asked to describe the percentage of their practice list that were young men under 25 years, and to reflect on the frequency of the visits these young men may make over a 12 month period, it often prompted GPs to reassess their perceptions of men’s low attendance, whilst PNs continued to assert that they had few opportunities to interact with young men.

I: ok. In terms of the proportion of young people on the list, would you say it’s kind of high or low?

R: Yes, we’ve got a lot of young people. [pause]. Hmm, yeah, I mean I suppose yeah they are here. (GP2, Male, SIMD5, 9.6%)

I: OK, so you have about 1 in 10 on your list are young men.

R: As much as that? But I would say personally I can’t remember the last time I gave a guy a bottle, probably about six months ago, a urine test for chlamydia.

I: Mm-hmm, mm-hmm. Do you see many young men at all?

R: No, no.

(PN5, Female, SIMD2, 10.5%)

Experiences of screening for chlamydia were strongly linked to the nature of the patient-led consultation, with many believing it easier to raise issues of sexual health with patients when attending for related issues. There was a common belief from both GPs and PNs that women are exposed more, or are used to, health-related messages pertaining to sexual and reproductive health as well as being more used to routine screening (e.g., cervical screening).

I think women are easier to talk to about things like that, especially younger women, and especially you’ve got them in for things like smears and stuff, you know, and sometimes when they come in for things like that they tend to open up a bit more about other things, especially to a woman who again they can maybe relate to being a bit like their mum, if you see what I mean! [laugh] (PN6, Female, SIMD5, 12.8%)

Many GPs described their reluctance to initiate conversations around sexual health with men. Descriptions of such encounters by GPs were often characterised as ‘difficult’, ‘awkward’ and ‘challenging’. As a consequence, any tests they conducted with men were largely driven by the men self-reporting symptoms, which would then lead to STI conversations and investigation.

It can be a bit awkward. It’s sort of how you gauge it. (GP6, Female, SIMD1, 13.7%)

Fewer nurses offered such comments, perhaps reflecting their infrequent contact with young men. Such embarrassment and discomfort was not always a key factor in failing to raise the issue of screening with men, particularly for those based at practices in areas of higher deprivation, who were not confident that chlamydia was a high priority for their patients.

… most of the young men I see are not coming in for sore knees, they’re coming in for methadone prescriptions and often quite complicated consultations…(GP10, Male, SIMD2, 11.2%).

Both GPs and PNs spoke of being uneasy with ‘unsolicited health care intervention’ and with making health promotion ‘leaps’.

I'd go out of my way to avoid randomly bringing up new things because we've got enough staff to deal with it and I'm always running 15 or 20 minutes late anyway. The fewer new unsolicited healthcare intervention the better [laughs] and we've got all the QOF [Quality Outcomes Framework] stuff to do. We're already bugging people enough…(GP4, Male, SIMD5, 10.2%)

However, when probed, participants admitted they asked unsolicited questions about smoking or alcohol, including to patients seeking advice for sports-related injuries.

These ‘leaps’ were justified by GPs because they were part of the practices’ contractual issues and related to financial incentives. The fewer PNs who spoke about these issues were related to the infrequency with which they interacted with men in their practice, but, like GPs, they still spoke more generally of ever present time pressures within the practice environment.

I think it’s just that there’s so much else going on in general practice at the moment that, you know, sort of screening the young male population just isn’t on the agenda. (GP8, Female, SIMD5, 16.1%)

I: What do you think might be the barriers of such an approach?

R: The cost. [pause] And the QOF, I mean that’s increased our workload year on year since I started doing this job so one more little thing. (PN6, Female, SIMD5, 12.8%)

Participants spoke of chlamydia screening being higher in their practice when payments were offered but witnessing, and participating in, a subsequent reduced concern once there was no longer a financial incentive for the practice.

We used to do it [screening] a bit more when it was run by the Health Board…we would get a payment for every test done, so probably its dropped a bit since that was withdrawn. (PN2, Female, SIMD5, 6.5%)

Although this PN worked at an affluent and low percentage practice, in terms of men registered, there seemed to be a real focus on payment and time-concerns, and little attention paid to the low percentages of young men registered. Such a focus was mirrored in the views of a PN from an affluent and higher percentage practice. She reflected on this payment period and suggested that in her practice there were so few positive infections identified that the £10 payment per screen was ‘*quite a lot of money to be spent on health, to reassure somebody’* (PN3, Female, SIMD3, 12.2%). Thus, their views coalesced around similar issues: payments and time.

### Views towards proactive, Internet-based screening

No GP or PN dismissed this approach outright as unworkable or unrealistic. All spoke in favour of it, in general terms, but offered a variety of views towards the ways it could be successful and reach the targeted populations for a high uptake, and the perceived challenges to its success. Views ranged from it being “wholly appropriate” and “entirely the way to reach” young men through to the still supportive, but tentative, “potentially quite a clever idea” and “worthwhile exploring”. The unanimous support for the use of the Internet for screening was commonly borne of the belief that technologies fall within the domain of ‘youth’, and are thus entirely appropriate for this population.

I presume that like technology is maybe the right way forward with this. Because that’s, you never see a young person that does not have a mobile phone. (PN8, Female, SIMD2, 12.0%)

Two GPs spoke of the reduction of hours for GPs if a nation-wide service with funding was introduced, leading to a favourable view towards an Internet-based approach.

…certainly if it [screening] was done at health board level, well I think all GPs would be happy with it (laughs) because it would be…yeah, out of their hands. (GP8, Female, SIMD5, 16.1%)

### Barriers and facilitators

#### Design and recruitment facilitators

The facilitators of an Internet-based approach to screening young people for chlamydia identified by participants focused on ease of access and convenience, as well as the importance of anonymity and confidentiality.

The easier it is for them, the better, probably. The more convenient it is for them, the better. (GP3, Female, SIMD5, 42.9%)

Almost every participant spoke of the anonymous or confidential nature of an Internet-based screening approach as being vital if it is to appeal to young people. For PNs in particular, this was borne out of their reflections of the potential for no anonymity in GP attendance, in particular the potential to ‘bump into’ someone.

I think they’re [young people] always concerned about the anonymity of things and GP practice, you go the doctors and you bump into your next door neighbour or your mother’s friend… (PN8, Female, SIMD2, 12.0%)

Confidentiality issues were raised by around half of all participants in relation to the type of data that would be accessed from registers for this approach; it was acceptable for age and date of birth data to be accessed but not detailed medical records. Most made the point that registers are being used for screening programmes, such as for cervical and bowel cancer. Four GPs and two PNs pondered whether some people may get annoyed at receiving an unsolicited screening letter, which might have a knock-on effect to practices.

Six GPs and two PNs mentioned practical issues that would need to be considered for an Internet screening approach so as not to become barriers, including who sends screening invitation letters and the accuracy of address information for young people. One GP believed there would need to be a ‘*very small step between the screening invitation and actually being able to do the test’* (GP9, Male, SIMD4, 10.9%).

Most participants spoke with ease about targeting particular populations for health education or screening offers, often referring to examples within their own practice such as previous efforts to screen for chlamydia or to reach out to young smokers on their practice list. The approach of targeting particular sub-populations, based on age, was not questioned. Although one GP did question whether men may face scrutiny by partners relating to infidelity if there was a lack of understanding that all young men were being offered screening.

…if the young man lives with a partner, and if the partner sees ‘chlamydia screening’, she needs to be told that it’s purely screening, and not that her partner’s been cheating around, and someone has asked for the partner to be tested, in case he’s got an infection because of his infidelity. (GP7, Male, SIMD1, 14.0%).

This underscores the importance of ensuring these processes are thought through carefully if they are not to become barriers to screening.

#### Socio-cultural barriers

Participants were often keen to stress that an Internet screening approach could be successful if young people considered testing as a normal thing to do. Half of all participants believed that normalisation of testing could be assisted by a nation-wide marketing campaign to kick start it, but also the need for such a campaign to continue so as to help keep momentum by keeping the service in young people’s minds.

…we didn’t have a screening process for cervical cancer when I was younger. So the first time I had one here I was like, oh why am I getting this? But now it’s…I expect it every three years and it’s not something that fazes me when it comes through the door. So again I think it’s… it would then become a bit more engrained that this is part of your health like having your blood pressure checked and things like that. (PN7, Female, SIMD5, 13.9%)

For some, such a widespread awareness of the screening taking place for all age-eligible young people may lead to relationships not becoming jeopardised by the screening letter arriving in the post.

Participants also stressed perceived barriers pertaining to gender-related issues, including perceptions among young people that chlamydia is a ‘woman’s disease’, associated with infertility and promiscuous women. Consequently, these participants believed that such young people fear the stigma of attending for a STI test where they can be seen and identified as promiscuous. No participant spoke about men in this way, but some did mention the embarrassment men may feel asking for a STI test. Support for the Internet approach therefore rested on the anonymity of the approach and non-clinic attendance. Young men were described as reluctant in general to discuss issues relating to their sexual health, although some went on to widen their thoughts on this to the issue of youth’s low perception of risk for STIs.

…the ostrich sort of thing - let's not think about it. it'll not happen, sort of thing (PN8, Female, SIMD2, 12.0%).

Participants also described women as more likely to be at ease with screening offers, given their experiences of cervical screening, but also with other regular medical intrusions in their lives due to contraception appointments. Reproductive health conversations were perceived to occur more often with women, and as such respondents questioned whether an approach that included men without an accompanying educational element might not ultimately reach men.

if you’ve got somebody who’s already got their awareness raised, and who’s thinking, “I probably ought to get this screening done, but I’m too embarrassed to go and talk to a GP about it.” If you’ve got somebody in that situation then, obviously, I think doing it on the Internet would be good. (GP10, Male, SIMD2, 11.2%).

## Discussion

This study obtained the views of GPs and PNs in Scotland towards Internet-based screening and assessed levels of support for the development of proactive screening targeting young heterosexual men via the Internet. The limitations include the small sample size and no male practice nurse being included in our sample, to allow for exploration of gender differences within PNs. Data were gathered from short but focused telephone interviews, which limit the richness of the available descriptions. We also recognise that, as is common across qualitative research, these views may not be representative of all GPs and PNs across Scotland, particularly those working at rural practices. Nevertheless, themes reflect those identified by GPs and PNs in other contexts [22], and provide valuable insights into the views of this group of health professionals concerning the acceptability and feasibility of this proposed intervention, should it become an approach to become implemented in a context with no current screening programme. The non-clinical background of the interviewer, made apparent to participants, acted to draw a fuller explanation from participants, who did not assume in-depth knowledge about primary care processes and policies, for example.

Our findings reveal the screening experiences of both GPs and PNs were largely with women, tied to beliefs that women simply consult more than men and that it is easier to raise sexual health issues within the type of consultations women are seeking, such as contraception or cervical screening. Screening men was low on the agenda, despite financial payment previously incentivising screening. Participants reported awkwardness and embarrassment in raising chlamydia screening with men, particularly if unrelated to the consultation; however, other unrelated health matters were often raised, tied to contracts, payment and workload. These findings are consistent with research conducted in England (where there is a National Chlamydia Screening Programme), particularly in relation to the barriers of raising sexual health with patients [22], and the role of financial incentives [24]. In the Scottish context, where there has never been a screening programme for chlamydia, the period of financial incentives failed to raise awareness of screening men, and certainly screening men was not maintained in any way after payment ceased. For men with no symptoms, they are unlikely to be offered an opportunistic test in this context. Removing the burden of screening from primary health settings by introducing an Internet-based approach could negate the need to return to the unsolved issue of training and payments by focusing the screening directly towards young men themselves.

Our findings reveal a high level of acceptability for an Internet-based screening approach, predicated partly on concerns for increased workload. A variety of views were offered concerning the ways it could be successful and reach the populations for a high uptake, as well as towards the perceived challenges to its success. The ease of access and convenience of this approach were identified by GPs and PNs as being facilitators. Anonymity and confidentiality, important issues raised by young people in other work [12,27,28], were also mentioned by participants as facilitators to an internet-based screening approach, as long as they were emphasised and assured to men. Barriers identified included the possible perception among men that they were being targeted on the basis of promiscuity or due to other perceived negative traits. Indeed, the issues raised by these health professionals were largely mirrored in the young men’s views towards Internet-based screening as a way to reach them: men wanted to be reassured that the approach would be easy, convenient and also confidential and were apprehensive about feeling targeted with screening [17].

The combined data from our study with GPs and PNs as well as with young men [17] have identified barriers and facilitators that would either help or hinder an Internet screening approach. We have identified support for the approach from these health professionals if screening is offered in a particular way, including being backed by a campaign to raise awareness. The young men provided a list of key ‘ingredients’ that would encourage their engagement with Internet-based screening, including: use a serious not a jokey tone; convey information simply; have an authentic voice by avoiding adults masquerading as youth, and; avoid fear narratives [17]. Identifying design-related and other barriers facilitators, from the target group as well as the health professionals who could support it, has been an important first step towards developing an Internet screening intervention, and follows the guidance on developing complex interventions [29]. Key findings to influence the intervention development include serious consideration to: guaranteeing confidentiality throughout, to ensure this does not end up a barrier to screening but instead acts as a facilitator; the introduction of an accompanying media campaign to raise awareness; engagement with health professionals who may have workload concerns and low perceptions of the priority of chlamydia screening (since it is no longer part of payments or key issues to raise with patients in the Scottish context); and further education and training for health professionals to counter awkwardness in discussing sexual health issues with men in unrelated consultations. These professionals also broadly supported the use of central registers to identify potential intervention participants (similar to cervical screening), which suggests this would not pose a major barrier to the implementation of our intervention. However, our data also suggest that any future involvement by health professionals may need to involve a financial incentive, which appears to have, to some extent, worked in the past to drive up screening among women. The intervention as envisaged does not include GPs and PNs at the level of testing, but could impact on workload if positive patients seek follow-up at their general practice; therefore, how any financial payment could work requires significant further consideration.

It is important to recognise that there are broader, key challenges in moving forward with further work on the use of the Internet for chlamydia screening, which pertain to the overall effectiveness of chlamydia screening as well as wider social and cultural factors associated with young people’s sexual behaviours. Concerns have been raised about the ability of screening to reach sufficient numbers of young people to reduce chlamydia prevalence and also for screening to interrupt subsequent sequelae, such as pelvic inflammatory disease [30]. The Dutch trial of a systematic approach to screening using the Internet, showed insufficient levels of uptake sustained over the three year screening period to reduce chlamydia prevalence [7]. Thus, reaching the target group, engaging them in screening and doing so repeatedly over time remains a key challenge. This is where the evidence of wider social and cultural barriers associated with young people’s sexual behaviours identifies issues that could continue to impede screening reach. These health professionals were cognisant of these barriers, even amongst themselves, which is now seen across multiple studies: a systematic review of qualitative literature on factors shaping young people’s behaviours found seven key themes across 268 studies, including social expectations impeding communication about sex [31].

## Conclusions

Our findings suggest that health professionals within primary care support an Internet-based screening approach and would support the use of registers to facilitate a proactive approach to reaching young men. They support the need to reach young men and recognise their own inability to engage men in screening, due to time pressures, lack of financial incentive and discomfort with raising screening with men. We also now require more than just increasing access to testing and communicating the ease of testing if greater screening coverage among key groups is to be achieved. An Internet approach to screening may not be able to tackle all of these issues, if it is only focused on the delivery of a service; therefore, the limits of this as a screening approach must be acknowledged. Internet-based approaches are acceptable to young people and professionals [15-17], and are reaching men and those from low socio-economic areas [21]. However, this approach requires accompanying efforts to tackle wider social and cultural barriers. What is required is to perhaps employ such approaches within a multi-faceted approach and ensuring effective interventions reach the policy agenda [32]. This would involve, for example, clinic-based testing and screening, a media and/or social marketing campaign to raise awareness, Internet-based screening, and self-testing via postal kits made freely available in shops, education and other settings. Within this, it remains clear that health professionals in primary care setting, such as those included in our work, may require further education and training within such a multi-faceted approach.

## Competing interests

The authors declare that they have no competing interests.

## Authors' contributions

KL and LM designed the study. KL collected the data; SM conducted the detailed coding and analysis, with input from KL and LM. KL wrote a first draft of the manuscript, collated comments from LM, SM and Prof Paul Flowers, thereafter re-drafting the manuscript. All authors approved the final manuscript.

## Pre-publication history

The pre-publication history for this paper can be accessed here:

http://www.biomedcentral.com/1471-2296/15/127/prepub
